# Preventative and Therapeutic Potential of Flavonoids in Peptic Ulcers

**DOI:** 10.3390/molecules25204626

**Published:** 2020-10-11

**Authors:** Wenji Zhang, Yingyi Lian, Qiuhua Li, Lingli Sun, Ruohong Chen, Xingfei Lai, Zhaoxiang Lai, Erdong Yuan, Shili Sun

**Affiliations:** 1 Guangdong Academy of Agricultural Sciences or Guangdong Key Laboratory of Tea Resources Innovation & Utilization, Tea Research Institute, Guangzhou 510640, China; zhangwenji@gdaas.cn (W.Z.); liqiuhua@tea.gdaas.cn (Q.L.); sunlingli@tea.gdaas.cn (L.S.); chenruohong@tea.gdaas.cn (R.C.); laixingfei@tea.gdaas.cn (X.L.); laizhaoxiang@tea.gdaas.cn (Z.L.); 2 School of Food Science and Engineering, South China University of Technology, Guangzhou 510641, China; 201820124821@mail.scut.edu.cn

**Keywords:** flavonoids, peptic ulcer, gastroprotective effects, antioxidation, anti- inflammation, antibacterial

## Abstract

Peptic ulcer disease is a common gastrointestinal tract disorder that affects up to 20% of the population of the world. Treatment of peptic ulcer remains challenging due to the limited effectiveness and severe side effects of the currently available drugs. Hence, natural compounds, owing to their medicinal, ecological, and other safe properties, are becoming popular potential candidates in preventing and treating peptic ulcers. Flavonoids, the most abundant polyphenols in plants, exhibit gastroprotective effects against peptic ulcer both in vivo and in vitro. In this review, we summarized the anti-ulcer functions and mechanisms, and also the bioavailability, efficacy, and safety, of flavonoid monomers in the gastrointestinal tract. Flavonoids exerted cytoprotective and rehabilitative effects by not only strengthening defense factors, such as mucus and prostaglandins, but also protecting against potentially harmful factors via their antioxidative, anti-inflammatory, and antibacterial activities. Although controlled clinical studies are limited at present, flavonoids have shown a promising preventable and therapeutic potential in peptic ulcers.

## 1. Introduction

A peptic ulcer is characterized as a mucosal break induced by acid or pepsin secretion in the gastrointestinal tract, especially the stomach and proximal duodenum [1]. Furthermore, peptic ulceration infiltrates through the mucosa layer to induce mucosal lesions, resulting in inflammation of the digestive tract [1,2]. Until the second half of the 20th century, *Helicobacter pylori* (*H. pylori*) infection and use of non-steroidal anti-inflammatory drugs (NSAIDs) were found to be the main risk factors of peptic ulcer [3]. Subsequently, various conditions, such as ischemia, inflammatory bowel disease (IBD), and renal diseases, and poor lifestyle, including stress, smoking, and excessive consumption of caffeine or alcohol, were also found to be risk factors of peptic ulcer [4]. Recently, a high incidence rate of 20% has been reported on peptic ulcers, which are mainly observed in 30–60-year-old people [4]. While the mortality rate of peptic ulcer is low, it is becoming prevalent and causes pain and severe complications. Peptic ulcer patients usually suffer from epigastric pain, such as burning or gnawing, and typical dyspeptic symptoms, such as bloating, nausea, fullness, and heartburn. Alternatively, some patients may experience complications, such as bleeding, perforation, and gastric outlet obstruction [5]. Among these, hemorrhage is the most frequent complication with increasing incidence that is up to 15%, which can be life-threatening [6]. The perforation often takes place and it makes patients experience intense pain in the abdominal area. Furthermore, swelling and scarring cause the duodenum to narrow, which can lead to gastric outlet obstruction. Under these circumstances, patients may experience severe vomiting or even vomit blood [7]. It can be seen that the complexity of this disease greatly affects the life quality of patients and also makes the development of effective and safe drugs very critical.

Treatment of peptic ulcer involves relieving pain, healing ulcers, and preventing further complications. Introduction of histamine (H_2_)-receptor antagonists, such as famotidine, cimetidine, and nizatidine, and proton pump inhibitors (PPIs), such as omeprazole, lansoprazole, pantoprazole, esomeprazole, and rabeprazole, for management of peptic ulceration has revolutionized the treatment options for peptic ulcer [8]. Antibiotic medications for treating peptic ulcer include amoxicillin, clarithromycin, metronidazole, tinidazole, tetracycline, and levofloxacin. Prostaglandin analogs, such as misoprostol, and cytoprotective agents, such as sucralfate, are also available for treatment of peptic ulcer. However, some remedies have side effects such as diarrhea, constipation, fatigue, drowsiness, headache, muscle aches, and acute liver injury [9]. For example, in some cases, cimetidine and ranitidine may cause idiosyncratic forms of hepatotoxicity [10,11,12]. Moreover, in case of acute complicated peptic disease and chronic complicated peptic ulcer disease, therapies using these drugs may be restricted [5]. Furthermore, the development of drug tolerance and incidence of relapses of peptic ulcer make the efficacy of these approved drugs arguable. For example, with increasing prevalence of antibiotic resistance, the effectiveness of *H. pylori* eradication with the standard PPI-based triple therapy (consisting of a PPI and two antibiotics, such as clarithromycin plus amoxicillin or metronidazole) has fallen from over 90% to 70% in many countries [1,13,14].

**Figure 1:**
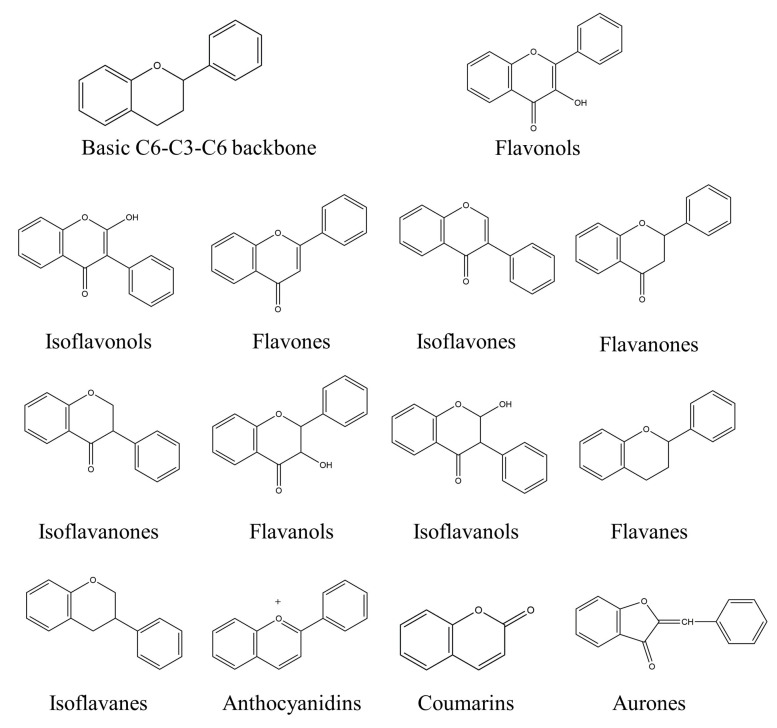
A basic structure of flavonoids.

Natural compounds found in diet and plants are generally used in such cases when drugs are to be used frequently or for chronic periods [15,16,17,18]. In recent years, an increasing number of studies have investigated natural compounds with gastroprotective effects, such as flavonoids, alkaloids, terpenes and terpenoids, saponins, phenolic acids, tannins, and fatty acids [19,20,21,22,23]. Of note, as one of the most abundant polyphenols in plants, flavonoids represent an important group of natural products that exhibit multiple pharmacological effects, such as antioxidative [24], anti-inflammatory [25], anticancer [26], antiviral [27], and anti-diabetic properties [28,29,30,31]. A large number of studies have demonstrated the protective effects of flavonoids on the intestinal epithelium [32,33,34,35], including maintaining intestinal barrier function, lipid and carbohydrate absorption, modulating enzyme activities, regulating the stomach of secretions, immune system regulation, and interaction with the pathogenic microorganism. All flavonoids have a basic C6-C3-C6 backbone structure and can be divided into 13 subgroups according to different substituents (Figure 1). Among these, flavonols, flavones, isoflavones, flavanones, flavanols, and anthocyanidins are particularly well-studied [30,36].

Here, we comprehensively searched reports on flavonoid monomers with anti-ulcer activity in the data banks of Scholar, PubMed, and Scopus and reviewed recent advances in flavonoids as a preventative and therapeutic treatment for peptic ulcer.

## 2. Anti-Ulcer Mechanisms of Flavonoids

**Figure 2:**
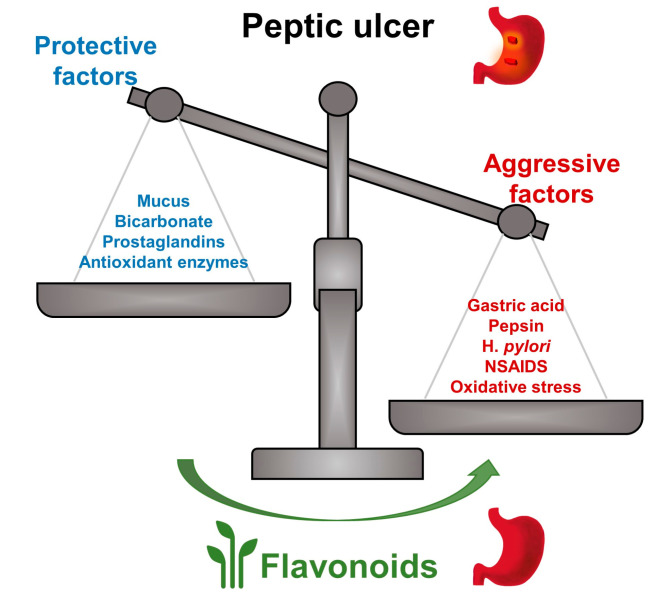
Flavonoids exert anti-ulcer effects through balancing protective factors and aggressive factors. Flavonoids show anti-ulcer effects by strengthening protective factors (mucus, bicarbonate, prostaglandins, antioxidant enzymes, etc.) and by resisting aggressive factors (gastric acid, pepsin, *H. pylori*, non-steroidal anti-inflammatory drugs (NSAIDs), oxidative stress, etc.).

Peptic ulcer is caused by an imbalance in gastrointestinal defense factors, such as prostaglandins, mucus, and bicarbonate, and potentially harmful factors, such as pepsin, acid, and *H. pylori* infection (Figure 2). Anti-ulcer effects of flavonoids include functions such as anti-acid secretion, inhibition of pepsin level and activity, and increasing gastric mucus and bicarbonate secretion. Additionally, flavonoids boost mucosal cytoprotective, antioxidative, anti-inflammatory, and antibacterial defenses against peptic ulcer. Usually, one type of flavonoid can exhibit anti-ulcer roles through multiple mechanisms.

### 2.1. Flavonoids Exert Anti-Ulcer Effects by Regulating Gastric Secretion Pathways

**Figure 3:**
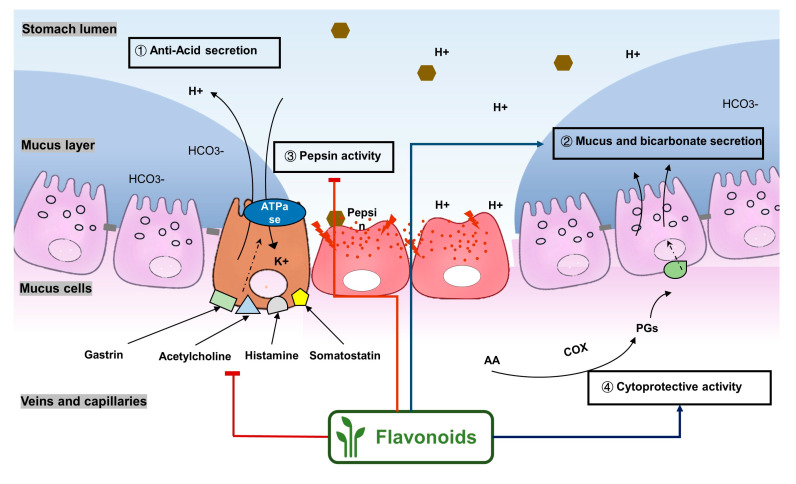
Flavonoids exert anti-ulcer effects through regulating gastric secretion pathways and prostaglandin levels. Flavonoids (**1**) decrease acetylcholine, gastrin, histamine, and somatostatin levels and inhibit H+K+-ATPase activities, therefore inhibiting gastric acid secretion; (**2**) promote mucus and bicarbonate secretion; (**3**) inhibit pepsin activity; (**4**) exhibit cytoprotective activity by regulating prostaglandin levels.

Normally, the stomach secretes a number of molecules, including gastric acid, pepsin, and gastric mucus. Stomach acid and pepsin promote digestion of ingested foods and gastric mucus protects the epithelial cells from damage due to gastric acid and pepsin [37] (Figure 3). However, a high concentration of gastric acid aggravates mucosal damage in peptic ulcer [38]. Therefore, inhibition of gastric acid excessive secretion is essential in peptic ulcer treatment. Gastric acid secretion is regulated by gastrointestinal hormones. Acetylcholine, gastrin, histamine are the main hormones that stimulate parietal cells to secrete acid. Additionally, somatostatin inhibits acid secretion and exerts a tonic restraint on parietal, enterochromaffin-like, and gastrin cells via acting on sst_2_ receptors [38,39]. More importantly, in the final step of gastric acid secretion, H^+^K^+^-ATPase, a proton pump in the membrane of parietal cells, catalyzes H^+^ transport at the expense of ATP hydrolysis. 

Flavonoids could exert anti-ulcer effects by inhibiting gastric acid secretion, similar to how histamine (H_2_)-receptor antagonists and PPIs work. Catechins, the most abundant polyphenol in tea, showed gastroprotective effects by regulating gastric secretion pathways. Wistar rats treated with 0.1% and 1% crude catechin for 2 weeks had a reduced gastric lesion index (from 23 ± 9 to 16 ± 5 and 9 ± 7, respectively) in a water immersion restraint stress model and inhibited the release of gastrin (from 108 ± 21 to 56 ± 12 and 46 ± 9 pg/dL, respectively), somatostatin (from 169 ± 23 to 74 ± 11 and 70 ± 25 pg/dL, respectively), and histamine (from 139 ± 21 to 92 ± 18 and 79 ± 19 nmol/L, respectively) in an isolated rat stomach infusion model [40]. It assumed that catechin might confer a protective effect by regulation of gastrointestinal hormones. However, caution is required for the decrease in somatostatin as it may lead to an increased acid amount to some extent. In ischemia reperfusion-induced gastric ulcer rats, administration of 50 mg/kg catechins for 3 days reduced the level of H^+^K^+^-ATPase from 1.15 ± 0.05 to 0.51 ± 0.03 mmol Pi liberated min^−1^ (mg protein)^−1^ and increased the plasma histamine level when compared to the model group [41]. Quercetin is a common flavonol that exists in the flowers, leaves, and fruits of many plants, such as *Quercus iberica* and *Dysosma veitchii*. Naringenin is a flavanone mainly found in grapefruits (*Citrus paradise*). Martin et al. [42] found that quercetin (100 mg/kg) and naringenin (100 mg/kg) both showed antihistamine and anti-ulcer effects in cold restraint-induced acute gastric ulcer and pylorus-ligate rat models but did not affect acidity and pepsin levels. Quercetin was also found to decrease histamine levels in gastric tissue in ethanol-induced gastric ulcer in rats at the dose of 200 mg/kg [43]. The main flavonoid from berries and red wine, myricetin, inhibited H^+^K^+^-ATPase activity with an IC_50_ value of 0.58 μM in a freeze-dried tubulovesicles enzyme assay; meanwhile, in an in vivo study, oral administration of 50 mg/kg myricetin attenuated histamine-induced gastric acid secretion in mice [44]. Methanolic extract from leaves of *Solidago chilensis* (Brazilian arnica) (100 and 300 mg/kg) and its flavonoid components, quercitrin (1.38 mg/kg) and afzelin (0.026 and 0.078 mg/kg), reduced the gastric lesion area caused by ethanol/HCl. Quercitrin and afzelin were proved to inhibit H^+^K^+^-ATPase activity by up to 30% and 33%, respectively [45]. Sofalcone is a synthetic derivative of sophoradine, an isoprenyl chalcone from *Sophora subprostrata* root. Chalcone, sofalcone, and sophoradine were found to inhibit pig gastric mucosa H^+^K^+^-ATPase activity in a dose-dependent manner. Kinetic studies suggested that sofalcone inhibited H^+^K^+^-ATPase competitively with ATP to block its phosphorylation [46]. These studies proved that flavonoids regulate gastrointestinal hormones and inhibit H^+^K^+^-ATPase activity, which are beneficial to inhibit gastric acid secretion and prevent further damage.

Flavonoids were also found to reduce the gastric acidity in peptic ulcer. Hesperidin, an abundant flavonoid in citrus fruits, was found to increase the pH and reduce the total acidity of gastric juice significantly (*p* < 0.001) at doses of 150, 300, and 450 mg/kg but only reduced the ulcer index at the dose of 450 mg/kg in the indomethacin-induced gastric ulcer rats. In a hypothermic restraint stress-induced gastric ulcer model, 300 and 400 mg/kg hesperidin both increased the pH value and reduced the total acidity of gastric juice and reduced the ulcer index significantly [47]. Another study showed that administration of 100 mg/kg hesperidin daily for 8 weeks decreased the gastric free acidity by 44% and the total acidity by 42%, increased the pH by 252%, and reduced the gastric ulcer index by 70% in a cold restraint stress-induced acute gastric ulcer model in diabetic rats [48]. Hypolaetin-8-glucoside, a flavonoid found in *Sideritis leucantha,* reduced the H^+^ concentration but not acid output and showed gastroprotective effects in both ethanol- and acetylsalicylic acid-induced gastric ulcer models of rats at the doses of 200 and 300 mg/kg [49]. O-methyl-3(+)-catechin, known as meciadanol, significantly reduced gastric acid output and concentration in a pylorus-ligated model at the dose of 150 mg/kg (*p* < 0.01) [50].

Besides gastric acid, pepsin is another endogenous aggressor in gastric juice. Excessive pepsin may cause extensive mucosal damage characterized by focal areas of discontinuity in the adherent mucus gel layer, punctate ulcers, and bleeding to lumen with no signs of re-epithelialization or mucus cap formation [51]. Hydroalcoholic extract of nettle leaves (*Urera baccifera*) exhibited gastroprotective effects in an ethanol-induced gastric ulcer model and decreased pepsin activity in the gastric juice in pylorus-ligated rats, and therefore, the flavonoids diosmetin and apigenin glucuronide were presumed to play major roles [52]. Yamahara et al. found that vexibinol from Sophora had anti-ulcer effects in various ulcer models, including HCl-ethanol, 0.6 N HCl, 0.2 N NaOH, absolute ethanol, and 1% NH_3_-induced gastric ulcer in Wistar rats. An amount of 300 mg/kg vexibinol administered intraduodenally inhibited acid and pepsin secretion significantly and had moderate effects on the pH value of gastric juice in pylorus-ligated rats [53].

Bicarbonate and mucus are regulated by prostaglandin and protect gastric epithelial cells against acid and pepsin [37]. Bicarbonate creates a pH gradient with a near-neutral pH at epithelial surfaces in the stomach and duodenum and provides the first line of mucosal protection against luminal acid. The continuous adherent mucus layer is a barrier to luminal pepsin and protects the underlying mucosa from proteolytic digestion [51]. In view of the research on this aspect, the flavonoid hesperidin, administered at 3 and 10 mg/kg twice daily for seven days, reduced the ulcer area by 34% and 62%, respectively, and accelerated gastric mucosal healing by increasing mucus secretion in a chronic gastric ulcer rat model induced by acetic acid [54]. Oral administration of 50, 100, and 200 mg/kg catechins prevented ethanol-induced gastric ulcer by 49%, 70%, and 100%, respectively, and increased the gastric hexosamine content by 12%, 44%, and 73%, respectively, which suggests that catechins may primarily protect gastric mucosa by gastric mucus-increasing actions and gastric mucosal hexosamine content-maintaining in ethanol-induced acute gastric mucosal injury rats [55]. Isoliquiritigenin, a chalcone found in licorice (*Glycyrrhiza glabra*), also promoted gastric mucus production in indomethacin-induced ulcer in mice at the dose of 100 mg/kg [56]. Pretreatment with the flavone chrysin (50 and 100 mg/kg), found in honey, propolis, and various plants, promoted mucus secretion and prevented acid production in a indomethacin-induced gastric ulcer rat model [57]. The flavonoid 2’, 4’-dihydroxychalcone, at the dose of 10 mg/kg, prevented the formation of gastric mucosal lesions to reinforce the mucosal barrier in water-immersion stress, acetic acid, and HCI/ethanol-induced gastric ulcer in Sprague-Dawley rats [58].

### 2.2. Flavonoids Show Gastric Cytoprotective Activity by Regulating Prostaglandins Levels

Prostaglandins (PGs), such as prostaglandin E2 (PGE2), are the main arachidonic acid metabolites. PGs regulate production of gastric mucus and bicarbonate and reduction in acid output, restore the gastric mucosa by dilating vessels, improve mucosal blood flow, and accelerate mucosal healing [59,60]. Two isoforms of cyclooxygenase (COX), cyclooxygenase-1 (COX-1) and cyclooxygenase-2 (COX-2), are key enzymes in the biosynthesis of PGs. The COX-1 isoform is expressed in most tissues, including the gastrointestinal tract, and produces PGs. In contrast, COX-2 has no or little expression in most tissues but is rapidly induced in the inflammatory setting. Usually, traditional NSAIDs, such as indomethacin, non-selectively inhibit both COX-1 and COX-2 and cause peptic damage with a marked decrease in gastric PGE2 content [61].

Flavonoids show gastric cytoprotective activities by regulating PGs’ biosynthesis pathways (Figure 3). From the studies, Alcaraz et al. [49] suggested that the gastroprotective effects of the flavone derivative hypolaetin-8-glucoside against the NSAID indomethacin could be involved in the cytoprotective effects of endogenous PGs. This flavonoid also increased the COX activity in in vitro experiments. A 48 mg/kg indomethacin treatment significantly decreased PGE2 level by 85.5% in Sprague-Dawley rats. By pretreatment with 50 and 100 mg/kg chrysin, the gastroprotective PGE2 level increased by 87% and 90%, respectively [57]. Isoliquiritigenin, mentioned above, not only decreased the gastric lesion area and increased the gastric mucus secretion but also increased gastric COX-2 expression in indomethacin-induced ulcer in mice. However, the gastric PGE2 content was not evaluated in the study [56]. Genistein, a soy-derived isoflavone, also increased the gastric PGE2 level to 210.3 ± 5.4 ng/g tissue when compared to the indomethacin treatment model group (113.3 ± 4.6 ng/g tissue) [62]. By administration of 100 mg/kg diosmin (a flavonoid abundant in citrus fruits), the PGE2 level was significantly increased when compared with the ethanol treatment group, despite ethanol also evoking depletion of PGE2 [63]. Nobiletin, a polymethylated flavonoid from citrus fruits, exhibited a gastric cytoprotective effect through regulating PGE2. Pretreatments with 10 and 20 mg/kg nobiletin both increased the serum PGE2 level significantly in an ethanol-induced gastric model in mice [64]. Furthermore, treatment with 0.02, 0.07, and 0.21 g/d licoflavone from *Glycyrrhiza* upregulated the levels of arachidonic acid and PGE2 to protect the gastric mucosa and accelerate mucosal healing in an acetic acid-induced gastric ulcer model [65]. 

### 2.3. Antioxidant Properties of Flavonoids in Peptic Ulcer

In the pathogenesis of peptic ulcer, reactive oxygen species (ROS), including superoxide anion radical (O_2_^−^), hydrogen peroxide (H_2_O_2_), and hydroxyl radical (OH), play an important role [66]. These species are normal byproducts of cellular metabolism, such as mitochondrial oxidative phosphorylation. When ROS concentration exceeds an organism’s antioxidative capacity, cells enter an oxidative stress state, in which ROS cause oxidative damage to cellular components. They lead to lipid peroxidation and damage cell membranes, resulting in the release of intracellular components and tissue damage. ROS also cause degradation of gastric epithelial base membrane components and change intracellular metabolism and DNA damage [67,68,69].

As potent antioxidants, flavonoids scavenge free radicals and decrease their formation, thus providing positive effects against peptic ulcer. Early studies showed that ternatin, a tetramethoxy flavone isolated from *Egletes viscosa* Less, could act against gastric mucosal damage induced by ethanol but not indomethacin or hypothermic restraint stress after pretreatment at concentrations of 25 and 50 mg/kg in rats, indicating that ternatin affords a gastroprotection effect through a PGs-independent mechanism, probably involving free radical scavenging and anti-inflammatory actions [70]. In one in vitro experiment, catechins (10^−5^–10^−1^ g/100 mL) developed O_2_^−^ scavenging activity in a concentration-dependent manner. In vivo, oral administration of catechins in rats at doses of 50, 100, and 200 mg/kg reduced ethanol-induced gastric mucosal injury by 49%, 70%, and 100%, respectively. Higher doses of 300 and 400 mg/kg catechins reduced stress-induced gastric injury by 80% and 93%, respectively [55]. In a H_2_O_2_/NaOH/DMSO-generated ROS system, compared to DL-α-tocopherol, a common natural antioxidant, garcinol, a flavonoid isolated from *Garcinia indica*, had a stronger effect in scavenging hydroxyl radicals, a weaker activity in scavenging methyl radicals, and a comparable ability in scavenging superoxide anions. Furthermore, oral administration of 200 mg/kg garcinol prevented acute gastric ulceration by radical formation induced by both indomethacin- and water-immersion stress [71]. Pretreatments for 24 h with 25 and 50 μM of quercetin, also known as 3,5,7,3′,4′-pentahydroxy flavone, both attenuated the increase in H_2_O_2_-induced oxidative stress in GES-1 cells. Quercetin also reduced gastric ROS accumulation in ethanol-induced gastric ulcer in Balb/c mice. Images of mice injected with ROS-sensitive L-012 were used to show the oxidative stress state of different groups. Obvious chemiluminescence signals were observed in the gastric region of mice treated with ethanol, but only weak chemiluminescence signals were detected after pretreatment with quercetin, suggesting that quercetin alleviated gastric ROS accumulation in ethanol-induced gastric injury [72].

Flavonoids not only scavenge ROS directly but also protect and activate antioxidant enzymes which, in turn, protect against oxidative damage in peptic ulcer. Antioxidant enzymes, such as superoxide dismutase (SOD), catalase (CAT), and glutathione peroxidase (GPx), combat free radicals and alleviate oxidative damage [73]. SOD catalyzes the highly reactive superoxide free radical (O_2_^−^) into less reactive hydrogen peroxide (H_2_O_2_) and molecular oxygen (O_2_), which is the first line of defense against ROS [74]. CAT breaks down H_2_O_2_ into water and molecular oxygen, consequently completing the detoxification process initiated by SOD [75]. GPx catalyzes the breakdown of H_2_O_2_ and inhibits lipid peroxidation [76]. Glutathione (GSH) is essential in maintaining gastric mucosal integrity, and depletion of GSH may induce mucosal ulceration [77]. It was found that in animal models of peptic ulcer induced by alcohols, stress, and NSAIDs, the activities of the antioxidant enzymes were decreased.

In an ischemia reperfusion-induced gastric ulcer model, pretreatment with 50 mg/kg (+)-catechins increased CAT and SOD activities (from 15.5 ± 1.3 to 32.4 ± 1.8 U, from 87.6 ± 12.4 to 145.0 ± 7.5 U, respectively) and reduced the level of malondialdehyde (MDA), an end-product of lipid peroxidation in peptic ulcer, from 0.48 ± 0.02 to 0.30 ± 0.01 nmol [41]. Catechins also showed gastroprotective effects in 95% ethanol-induced acute gastric ulcer by preventing the depletion of SOD activity and GSH level and by reducing lipid peroxidation at the doses of 25 and 50 mg/kg [78]. Quercetin accounted for the anti-ulcer ability of methanolic extract from *Madhuca indica* J. F. Gmel. (Sapotaceae) leaves. Treatment with 5 and 10 mg/kg quercetin for 14 days showed significant and dose-dependent healing effects on the ulcerated area that was caused by acetic acid in comparison to the control group in rats. At the same time, gastric SOD and GSH levels were also elevated significantly while the level of MDA decreased [79]. Treatment with low doses of rutin (20, 40, and 80 mg/kg), a flavonol of *Ruta graveolens*, reduced the ulcer index in all ethanol-, acetic acid-, and stress-induced ulceration models by increasing vitamin C and GPx activity and decreasing MDA levels [80]. In the 1,1-Diphenyl-2-picrylhydrazyl radical (DPPH) free radical scavenging assay, anthocyanins of *Rubus coreanus* exhibited free radical scavenging activities of 8.46%, 20.47%, 37.31%, and 69.17% at the doses of 10, 25, 50, and 100 μg/mL, respectively. In the naproxen-induced gastric ulcer model in rats, pretreatment with 20, 50, and 80 mg/kg anthocyanins twice daily for 3 days reduced gastric MDA levels and increased CAT and SOD activity [81]. Isoorientin, derived from *Eremurus spectabilis*, significantly decreased the MDA level (*p* < 0.05) and increased SOD activity and the GSH level in gastric tissues at doses of 25, 50, and 100 mg/kg [82]. Flavonoids, including hesperidin [47,48,54,83], diosmin [63], nobiletin [64], and genistein [62], aromadendrin-4′-*O*-methyl-ether and kaempferide [84] from Brazilian green propolis, and biochanin A [85] from soy and red clover also showed positive effects in treating peptic ulcer by increasing activities of antioxidant enzymes in vivo. 

Flavonoids can also regulate nuclear factor erythroid 2-related factor 2 (Nrf2)/heme oxygenase-1 (HO-1) pathway to improve oxidative stress. Nrf2 is a key transcription factor that regulates phase II detoxification and upregulates antioxidant genes HO-1. Upregulation of HO-1 expression results in increased accumulation of iron, bilirubin, and carbon monoxide, which in turn reduces the sensitivity of gastrointestinal cells to oxidative damage [86,87]. Treatment of Int-407 cells with 100 μM catechin decreased the ROS and lipid peroxidation, increased the activity of antioxidant enzymes, and upregulated nuclear/cytosol Nrf2 ratio and HO-1 protein expression in a time-dependent manner when compared with the ketoprofen-exposed model group. Sprague-Dawley rats pretreated with 35 mg/kg catechin for 21 days before the administration of ketoprofen also exhibited a reduced gastric ulcer area [88]. The flavonoid hesperidin was found to increase gastric expression of HO-1 and Nrf-2 in stress-induced gastric ulcer in diabetic rats [48]. Silymarin, a flavonoid mixture from the *Silybum marianum* (milk thistle) plant, prevented oxidative stress by enhancing GSH and SOD activities, upregulating the Nrf2 gene and inhibiting lipid peroxide production in indomethacin-induced gastric ulcer in albino rats [89].

**Figure 4:**
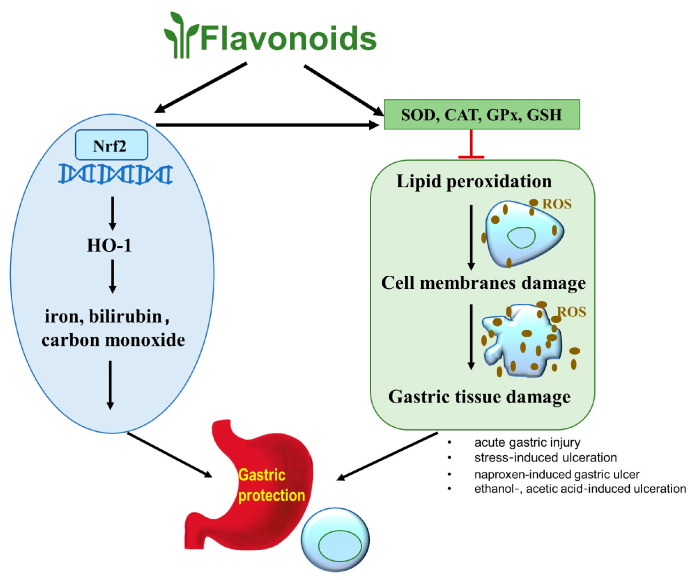
Flavonoids have beneficial effects on treating peptic ulcer via antioxidative activity. Flavonoids increase the activities of antioxidant enzymes, such as superoxide dismutase (SOD), catalase (CAT), glutathione peroxidase (GPx), and glutathione (GSH). and the nuclear Nrf2 level to exert gastroprotective effects through scavenging reactive oxygen species (ROS) and up-regulating the phase II detoxification and antioxidant genes HO-1.2.4 Flavonoids ameliorate peptic ulcer by regulating inflammatory pathways.

This section showed that flavonoids have beneficial effects on treating peptic ulcer via antioxidative activity (Figure 4). Flavonoids increase the activities of antioxidant enzymes SOD, CAT, GPx, GSH, and the nuclear Nrf2 protein level to scavenge ROS, then prevent the lipid peroxidation and protect the integrity of cell membranes and gastric tissue. Besides, the increased Nrf2 protein upregulates HO-1 to increase the iron, bilirubin, and carbon monoxide to minimize oxidative damage of gastric tissue.

Flavonoids ameliorate inflammatory symptoms in peptic ulcer by regulating myeloperoxidase (MPO), nitric oxide synthase (NOS), inflammatory signaling pathways, and inflammatory cytokines. 

MPO is considered as a biomarker of neutrophil infiltration and possesses pro-oxidative and proinflammatory properties [90]. In the development of peptic ulcer, recruitment of neutrophils and other inflammatory cells to the damaged sites activates secreted enzyme MPO, which promotes oxidative stress [91,92]. Flavonoids, such as quercetin [43], quercitrin and afzelin [45], genistein [62], aromadendrin-4′-*O*-methyl-ether and kaempferide [84], kaempferol [93], and rutin [94,95], have been shown to decrease MPO levels, thereby exhibiting anti-inflammatory activity in peptic ulcer.

Nitric oxide (NO) plays a multifaceted role in gastric mucosal stability. Low concentration of NO produced by constitutive nitric oxide synthase (cNOS) helps to retain gastric mucosal integrity, mediate gastric blood flow, and inhibit gastric acid secretion [96]. However, inflammation triggers upregulation of inducible nitric oxide synthase (iNOS) in macrophages and neutrophils, which increases NO levels and results in cytotoxic effects and gastric oxidative damage [96,97,98]. Pretreatment with rutin at doses of 50, 100, and 200 mg/kg exhibited gastroprotective effects against gastric ulcer induced by ischemia-reperfusion by preventing elevation of iNOS activity and by inhibiting cNOS and MPO activity in the gastric mucosa [94]. Indomethacin administration decreased the total nitrite/nitrate level in gastric mucosa, which may be related to a decreased production of cNOS in gastric tissue and upregulation of iNOS in neutrophils and macrophages. The catechin monomer epigallocatechin gallate (EGCG) showed ulcer-healing action against indomethacin-induced gastric ulcer by regulating NO levels. Treatment with 2 mg/kg EGCG reduced serum nitrite levels, suppressed the serum nitric oxide synthase activity, reversed increased iNOS expression, and reduced endothelial NOS expression in gastric tissues damaged by indomethacin [99].

Neutrophil infiltration leads to the production of pro-inflammatory cytokines, such as tumor necrosis factor-α (TNF-α), interleukin-6 (IL-6), and interleukin-1β (IL-1β), which is associated with the nuclear factor kappa B (NFκB) pathway and mitogen-activated protein kinases (MAPK) signaling cascades [100]. Stimulations such as ROS and NO or external stimuli activate NFκB by degrading iκB-α and phosphorylating NFκB p65/p50 subunits. Activated NFκB p65/p50 then translocate from the cytoplasm to the nucleus and promote gene expression of pro-inflammatory cytokines [101]. The MAPK cascade (ERK, extracellular-signal-regulated kinases; JNK, c-Jun *N*-terminal kinases; p38, p38 mitogen-activated protein kinases) is closely related to inflammation response. As mentioned above, COX-2 is induced primarily during inflammation and regulates the inflammatory response in gastric injury [102]. Pro-inflammatory cytokines, such as TNF-α and IL-1β, upregulate the expression of COX-2 via the NFκB pathway. Complicatedly, COX-2 not only plays roles in the production of PGs but also increases leukocyte adhesion and neutrophil activation, which aggravates peptic ulceration [103]. 

**Figure 5:**
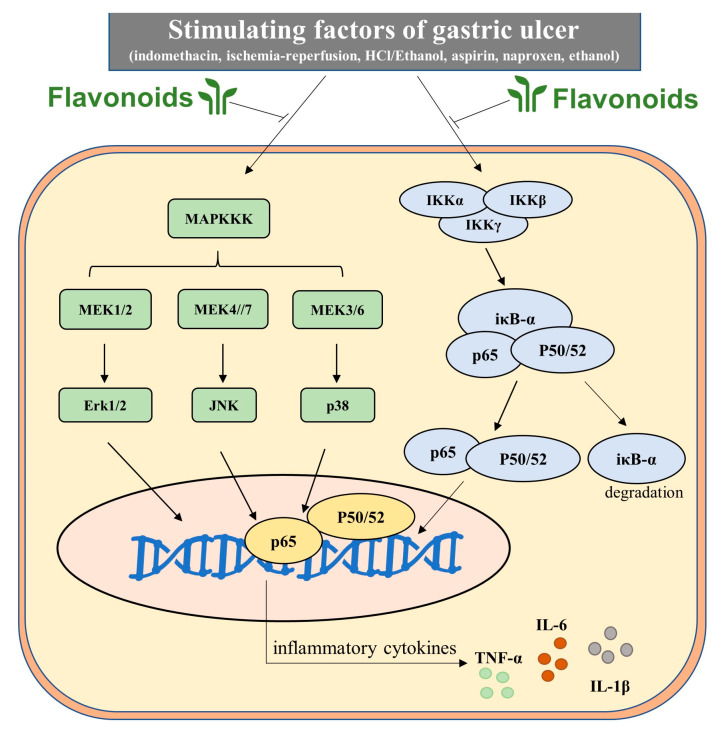
Flavonoids play key roles in inhibiting the occurrence and development of inflammation through the MAPK/P65 pathway by reducing inflammatory cytokine levels. In total, some factors activate MAPK upstream kinase and IKK complex (a, β, γ), MAPKKK activates downstream cascade MEK and Erk, JNK and P38 step by step. On the other hand, the IKK complex phosphorylates IκB proteins and frees NF-κB/Rel complexes to translocate to the cell nucleus. The activated MAPK downstream kinases and NF-κB p65/p50/52 would increase the expression of pro-inflammatory TNF-α, IL-1β, and IL-6 and augment anti-inflammatory IL-10. Flavonoids would regulate kinases involved in these two signaling pathways and inhibit pro-inflammatory cytokines to ameliorate inflammatory symptoms in peptic ulcer.

Diosmin was found to suppress gastric inflammation by reducing MPO activity and TNF-α and NF-κB levels. Levels of anti-inflammatory interleukin-10 (IL-10) were augmented by diosmin in ethanol-induced gastric ulcer in rats [63]. In the ethanol-induced gastric ulcer model, kaempferol, a flavanol from *Kaempferia galanga* L, decreased the plasma level of TNF-α by 33%, 43%, and 48%, and that of IL-1β by 46%, 43%, and 37% at doses of 40, 80, and 160 mg/kg, respectively [93]. Gastric injury induced by HCl/ethanol and upregulation of neutrophil infiltration triggered by aspirin were both ameliorated by oral administration of kaempferol (3 and 30 mg/kg) which decreased the levels of iκB, JNK, and p38 [104]. Hesperidin exhibited anti-inflammatory activities by reducing the gastric TNF-α and COX-2 levels in ethanol-induced peptic ulcer in rats compared to the model group after administration at 50 mg/kg for 15 days [83]. Anthocyanins from Korean blackberries (*Rubus coreanus*) have shown anti-gastric ulcer effects in association with the antioxidative and anti-inflammatory activity. In a naproxen-induced gastric ulceration rat model, oral administration of anthocyanins (20, 50, and 80 mg/kg b.w.) twice daily for 3 days attenuated the expression of pro-inflammatory cytokines TNF-α and IL-1β and activated the expression of metalloproteinase-2 (MMP-2), a zinc-dependent endoproteinase associated with inflammation [81]. Nobiletin, a major polymethoxyflavone in citrus fruits, reduced the MPO activity and pro-inflammatory cytokines via the MAPK pathway in ethanol-induced acute gastric ulcer in mice [64]. Chrysin [57], genistein [62], quercetin (3,5,7,3′,4′-pentahydroxy flavone) [79], and silymarin [89] also showed similar anti-inflammatory activity in peptic ulcer. Overall, these findings suggest that flavonoids may play protective roles in peptic ulcer by inhibiting inflammatory pathways (Figure 5).

### 2.4. Flavonoids Possess Anti-H Pylori Activities for Peptic Ulcer Healing

Bacteria *H. pylori* infection is the strongest known risk factor for peptic ulcer and even gastric cancer. This bacterium produces urease to maintain an alkaline environment for survival in acidic stomach. Urease catalyzes urea to ammonia which is toxic to intercellular junctions and causes tissue injury [105]. *H. pylori* expresses adhesins, such as blood group antigen adhesin (BabA) and outer inflammatory protein adhesin (OipA), which are needed for the attachment of these bacteria to the gastric epithelium [1,106,107]. *H. pylori* induces secretion of chemokines and pro-inflammatory cytokines, such as monocyte chemotactic protein-1 (MCP-1), IL-6, and TNF-α, which induces an inflammatory response in the early stage of infection [108]. *H. pylori* also induces oxidative burst in neutrophils recruited to the gastric injury sites and causes mucosal damage [109,110].

Both in vitro and in vivo studies suggested that many kinds of flavonoids possess anti-*H. pylori* activities, thus providing a benefit in peptic ulcer healing. Six tea catechins, including EGCG, epicatechin gallate, epigallocatechin, epicatechin, crude catechin (Polyphenon70SR), and crude theaflavins, possessed anti-*H. pylori* activities, with EGCG being the most active one. In vivo studies further proved that a Mongolian gerbil feeding diet containing catechins showed positive effects against *H. pylori* and protected the gastric mucosal [111]. After oral administration of 200 mg/kg quercetin for 15 days, a significant decrease of *H. pylori* infection was found in both the antrum and corpus mucosa in *H. pylori*-induced gastric ulcer in guinea pigs. Meanwhile, quercetin ameliorated inflammation and lipid peroxidation [112]. Moon et al. [113] tested anti-*H. pylori* activities of natural flavonoids by a paper disc diffusion test. The results showed that quercetin, kaempferol, naringenin, luteolin (flavonoids from *Resedaceae* plants), and hesperetin (flavonoid from the peels of *Citrus maxima*) inhibited the growth of *H. pylori*, and therein, 7-*O*-Butylnaringenin, a novel flavonoid modified from naringenin, was the most effective one. Moreover, 7-*O*-Butylnaringenin and hesperetin also inhibited the urease activities of *H. pylori*. In contrast, up to 20 mM of hesperidin, apigenin (a flavonoid rich in *Apium graveolensvar. dulce*, *Selaginella tamariscina,* and *Sabinachinenesis*), and genkwanin (also known as 4′5-dihydroxy-7-methoxyflavone from *Daphne genkwa* Sieb. et Zucc.) showed no effects on the growth of *H. pylori*. Fukai et al. [114] isolated 15 known flavonoids and 3 new isoflavonoids from licorice, a medicinal plant used for the treatment of peptic ulcer. Among these flavonoids, vestitol, licoricone, 1-methoxyphaseollidin, gancaonol C, glycyrin, formononetin, isolicoflavonol, glyasperin D, 6,8-diprenylorobol, gancaonin I, dihydrolicoisoflavone A, and gancaonol B exhibited anti-*H. pylori* activities in vitro. Ustün et al. [115] isolated three flavonoids, namely isorhamnetin (quercetin 3-methyl ether), quercetin 3,7-dimethyl ether, and kaempferol 3,7-dimethyl ether, from the chloroform extract of *Cistus laurifolius*. All of these three flavonoids showed in vitro anti-*H. pylori* activities. Isoflavone aglycones, irisolidone, tectorigenin, and genistein from flowers and rhizomes of Leguminosae (*Pueraria thunbergiana*) were also found to inhibit *H. pylori* growth in an in vitro study [116]. 

### 2.5. Other Mechanisms

Flavonoids also prevent or treat peptic ulcer by regulating amino acid metabolism, gastrointestinal motor activity, or other factors. One study showed that treatment with licoflavone caused a change in the content of amino acids, histidine, tryptophan, lysine, and glycine in plasma in acetic acid-induced gastric ulcer rats [65]. Intragastric administration of marmin and nobiletin from *Aurantii fructus immaturus* at a dose of 25 mg/kg significantly inhibited gastric motor activity, which could be helpful for gastric emptying during gastric ulcer [117]. Besides anti-acid secretory, cytoprotective, antioxidative, and anti-inflammatory activities, chrysin also showed gastroprotective effects by promoting angiogenesis by upregulating the expression of vascular endothelial growth factor (VEGF), basic fibroblast growth factor (bFGF), and adhesion molecule CD31 (platelet endothelial cell adhesion molecule-1) [57].

## 3. Alternative Strategies for the Treatment of Peptic Ulcer with Flavonoids

### 3.1. Combination Therapy of Flavonoids and Approved Drugs

In recent years, the combination therapy of flavonoids and approved drugs was an alternative strategy for the treatment of peptic ulcer. It is beneficial to overcome some disadvantages, especially drug resistance, when using antibiotics in treating *H. pylori* eradication [1,13,14]. Isomoto et al. studied the combination treatment of the flavonoid sofalcone and standard triple therapy in a clinical trial of 165 patients with peptic ulcer with *H. pylori* infection. Combination treatment of the flavonoid sofalcone (100 mg twice daily) and standard triple therapy with rabeprazole (10 mg twice daily), clarithromycin (200 mg twice daily), and amoxicillin (750 mg twice daily) for 7 days significantly improved the cure rate (94%) compared to the typical triple therapy without sofalcone (84.9%) and compared to the combination treatment of polaprezinc (anti-ulcer drug) and typical triple therapy (84.9%) using per protocol analysis [118]. The evidence also showed that pretreatment with a combination of famotidine and quercetin improved the gastroprotective effects in indomethacin-induced gastric ulcer in rats when compared to famotidine treatment alone. Famotidine is one of the most potent antagonists for peptic ulcer and it has rare side effects. However, its low oral bioavailability (40–50%) and short biological half-life (2–4 h) limit its efficacy [119]. Treatment with a combination of 12 mg/kg famotidine beads and 50 mg/kg quercetin significantly reduced the ulcer index and MPO level and prevented GSH, SOD, and CAT level decrease (*p* < 0.05) compared with the model group and the famotidine group alone [77]. 

Studies of combined drug–flavonoid therapies of peptic ulcer are still limited. However, a combination of drugs and flavonoids showed improved effectiveness in the peptic ulcer treatment, suggesting a novel treatment strategy for peptic ulcers. 

### 3.2. Bioavailability Improvement of Flavonoids on Peptic Ulcer

Poor bioavailability is one of the major limitation of flavonoids in both in vivo study and clinical application. For example, Choi et al. [56] studied the in vivo gastroprotective effects and pharmacokinetics, tissue distribution, and metabolism of isoliquiritigenin in mice. Due to the metabolism, the absolute bioavailability of isoliquiritigenin was low, but the absorbed fraction of isoliquiritigenin was high. One thing we want to emphasize is that isoliquiritigenin was highly distributed in the stomach in the tissue profiles. Considering the above problems and characteristics of flavonoids, some studies have explored new technologies to improve its efficiency.

Novel formulation strategies, such as nanoencapsulation technology, including liposomes, microspheres, and nanocapsules, have a great potential to improve bioavailability of flavonoids [4,120,121]. Polymeric nanocapsuled-quercetin had about a 20-fold higher efficacy than the free one in inhibiting the upregulation of the matrix metalloproteinase and infiltration of inflammatory cells and oxidative stress in ethanol-induced gastric ulcer in rats. It also reduced the gastric ulcer by 90%, which was better than the effects of famotidine (80%) [122]. Similarly, in the case of diosmin, the coated chitosan-poly (d,l-lactide-co-glycolide) (PLGA) nanoparticle version showed greater anti-ulcer activity by reducing the ulcer area and suppressing inflammation in ethanol-induced gastric ulcer than the free diosmin due to a prolonged residence time and better bioavailability [123]. Hence, nanoencapsulation technology would attract much more attention to enhance cell uptake of flavonoids with minimal systemic side effects in peptic ulcer treatment.

## 4. Safety Assessment of Flavonoids on Peptic Ulcer

The long history of flavonoid-daily intake (about 4 million years) of human beings reflects the safety of flavonoids through evolution [124]. The balanced diet including a daily intake of vegetables, fruits, or beverages containing a considerable amount of flavonoids, which keep us healthy, also proved the long-term safety of flavonoids [25,30]. Recent scientific research also suggested that natural flavonoids have a wide safety margin and hardly cause acute toxic effects [30,125,126], although we have no qualms about saying that it should be concerned that in some extreme circumstances, such as intravenous injection of a large amount of flavonoids could be dangerous [30,127].

From the following data, it is not difficult to find that the effective concentration of flavonoids is far lower than the concentration of its toxicity or side effects. Specifically, several studies assessed the gastroprotective effects and toxicity of flavonoid monomers in peptic ulcer. According to the guidelines of the Organization for Economic Co-operation and Development, the toxic class of hesperidin in Wistar rats was assessed and there was no toxicity sign during the 14-day observation, and the lethal dose of hesperidin was higher than 2000 mg/kg, which is far beyond the effective concentration of 300 and 400 mg/kg [47]. The toxicity and effectiveness of biochanin A were assessed by orally feeding rats using six dosages (from 250 to 5000 mg/kg). The behavioral, neurological, and autonomic behaviors of animals were under observation for 2 weeks without any sighs of diarrhea, weakness, tremors, seizures, or loss of controlled movement. There were no statistically significant differences of kidney and liver parameters (such as total protein, albumin, globulins, chloride, anion gap, potassium, sodium, urea, and creatinine) between the rats given vehicle 10% Tween 20 and rats given 250 and 5000 mg/kg biochanin A (*p* < 0.05) [85]. Following oral administration of 30, 300, and 3000 mg/kg/d quercetin for 28 days in Swiss mice, no histopathological changes were found in the organs and the biochemical variables were normal at any doses of quercetin [128]. Intraperitoneal administration of myricetin at an extreme dose of 1000 mg/kg did not cause any death of mice [125]. In acute toxicity studies conducted in Wistar rats, up to 500 mg/kg/d genistein was considered safe without any adverse effects [129]. In histamine-induced gastric acid secretion in mice, oral administration of 50 mg/kg myricetin attenuated gastric ulcer and caused no irregular behavioral symptoms [44]. 

In addition to flavonoid monomers, there are many toxicological studies on flavonoid-rich compounds, which also fully demonstrated the safety of flavonoids within a certain range. One acute toxicological study on grape seed extract (with proanthocyanins as the main component) showed that the oral dose of 4 g/kg body weight of grape seed extract had no physiological effect on rats [130]. Oral administration of 1000 mg/kg of rutin-rich (76 ± 3%) *Dimorphandra mollis* dry extract was considered safe in rodents by acute and chronic (180 days) toxicity evaluation [131]. When studying the gastroprotective effects of (+) -catechin hydrate on gastric ulcer induced by ethanol in rats, the safety of oral administration of 125 mg/kg and 250 mg/kg (+)-catechin hydrate was also assessed. Rats given the two dosages of (+) -catechin hydrate did not show any indications of toxicity during the 14-day observation. No marks of toxicity were shown in the liver and kidney either [78]. 

Though more clinical data are needed to prove the safety of flavonoids, the effectiveness of flavonoids at low concentrations showed that they have a high prospect in drug development.

## 5. Conclusions

Here, we listed a total of 60 kinds of flavonoids which exerted gastroprotective effects in different peptic ulcer models. Of note, except catechins, theaflavins, and anthocyanins with definite chemical composition and structure, there are 29 and 37 flavonoids monomers were displayed in prevention (Table 1) and treatment (Table 2) strategies, respectively. From the tables, we clearly found that tea, fruits, soy, licorice, and honey were the main sources of flavonoids. It is no surprise that catechins and their monomers from tea, one of the most popular drinks worldwide, have drawn most attention both in preventative and therapeutic treatment. Quercetin and its derivative also played important roles in the improvement of gastric ulcer. Moreover, the preventive effect of flavonoids on gastric ulcer received much more attention than the therapeutic research, and it has almost been verified through in vivo experiments. In recent years, more reports that flavonoids also have remarkable therapeutic effects on peptic ulcer have gradually emerged, although they were more often proved in vitro, which showed that flavonoids have a prospect in the treatment after injury. Per os (p.o.) and intragastric administration (i.g.) are the most frequently used methods at the concentration of 3~100 mg/kg range of flavonoids, which are far below the LD50 at the 2000 mg/kg in acute toxicity test discussed above. However, patients with peptic ulcer are underrepresented in clinic trials. At present, most data came from laboratory model tests. The tables clearly revealed that the human intestinal epithelial cell line (int-407 cells) and the human gastric mucosal epithelial cell line (GES-1 cells) were usually used as in vitro models to assess the protective effects of flavonoids. Moreover, the in vivo models of peptic ulcer include ulcers caused by oxidative damage, ethanol, NSAIDs, stress, and *H. pylori* or acid-ethanol (ethanol or ethanol/HCl)-induced acute gastric ulcer models. These models reflected the causes and phenotypes of human peptic ulcer disease, although the protective effects of the flavonoids in these models are often determined by the route of administration, animal species, duration, and the dose of administration. 

**Figure 6:**
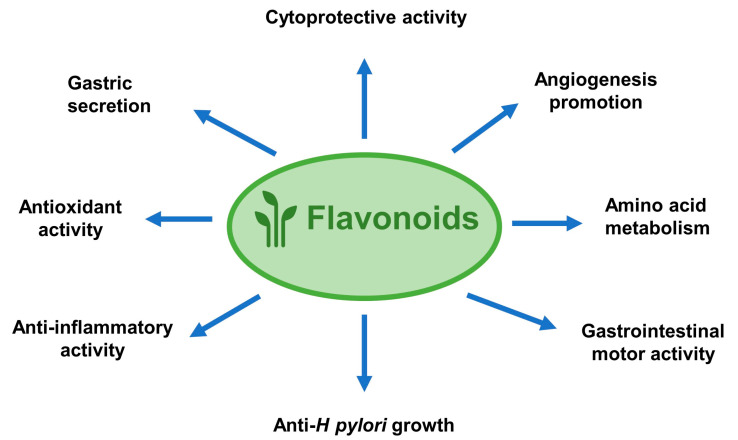
The anti-peptic ulcer effects of flavonoids have several mechanisms, including anti-acid secretory activity, cytoprotective effects, anti-oxidant activity, anti-inflammatory, anti-*H. pylori* growth, angiogenesis promotion, amino acid metabolism regulation, and gastrointestinal motor activity promotion.

Flavonoids are abundant secondary metabolites in nature with potentially beneficial effects on human health. These compounds have been shown to protect the gastrointestinal mucosa against ulcer in animal studies and in limited clinical trials. A combination of flavonoids and existing drugs or the nanoencapsulation of flavonoids were found to exhibit better therapeutic effects on peptic ulcer when compared to the single or standard treatment. Flavonoids exhibit several anti-ulcer protective mechanisms, such as anti-acid secretory activity, cytoprotective effects, antioxidative activity, anti-inflammatory, and antibacterial activity (Figure 6). Although future controlled clinical studies and bioavailability improvements are needed to assess the efficacy of flavonoids in preventing and/or treating peptic ulcer disease, it is still undeniable that flavonoids, especially the monomers, are suitable candidates in preventing as well as treating peptic ulcers.

## Figures and Tables

**Table 1:** Studies on the prevention of peptic ulcer by flavonoids.

Substance	Structure	Sources	Experimental Assay	Dose	Activity	Ref.
Catechins	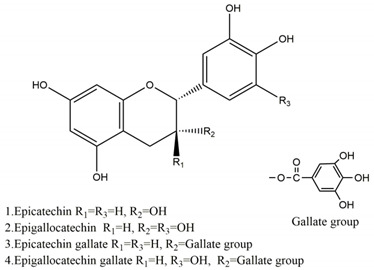	Tea	Water immersion restraint (WIR) stress-induced gastric mucosal lesion model and isolated rat stomach infusion model in Wistar rats	0.1% crude catechin-containing water (p.o.)	Active	[40]
			Absolute ethanol-induced gastric ulcer in Sprague-Dawley strain SPF rats	50 mg/kg (p.o.)	Inactive	[55]
				100 mg/kg (p.o.)	Active	
				200 mg/kg (p.o.)		
			Restraint plus water immersion stress in Sprague-Dawley strain SPF rats	100 mg/kg (p.o.)	Active	
			Ethanol-induced gastric ulcer in Sprague-Dawley rats	25 mg/kg (p.o.)	Active	[78]
				50 mg/kg (p.o.)		
			Ketoprofen-induced oxidative damage in the gastrointestinal mucosa in Sprague-Dawley rats	14 mg/kg (p.o.)	Active	[88]
				35 mg/kg (p.o.)		
			Ketoprofen-induced damage in humanInt-407cell line	100 μM (in vitro)	Active	
Quercetin	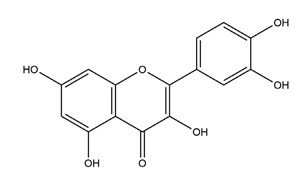	*Quercus iberica*, *Dysosma veitchii*	Cold restraint-induced gastric ulcer and pylorus-ligate induced gastric ulcer in Wistar rats	100 mg/kg (i.g.)	Active	[42]
			Ethanol-induced gastric ulcer in Sprague-Dawley rats	200 mg/kg (i.g.)	Active	[43]
			Ethanol-induced gastric ulcer in Balb/c mice;	25 mg/kg (p.o.)	Active	[72]
			H_2_O_2_-induced damage in GES-1 cells	6.25 μM (in vitro)	Inactive	
				12.5 μM (in vitro)		
				25 μM (in vitro)	Active	
				50 μM (in vitro)		
				100 μM (in vitro)	Inactive	
			Ethanol-induced gastric ulcer in Sprague-Dawley rats	Not mentioned	Active	[122]
Naringenin	* 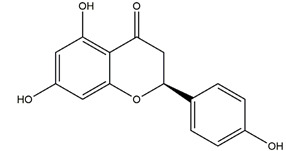 *	Grapefruits *(Citrus paradise)*	Cold-restraint induced gastric ulcer and pylorus-ligate induced gastric ulcer in Wistar rats	100 mg/kg (i.g.)	Active	[42]
Myricetin (3,3′,4′,5,5′,7-hexahydroxyflavone)	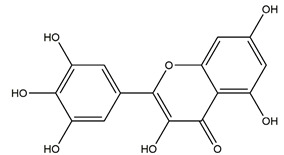	Berries and red wine	Enzyme assay using freeze-dried tubulovesicles prepared from hog stomach; histamine-induced gastric acid secretion in ICR mice	50 mg/kg (i.g.)	Active	[44]
Quercitrin	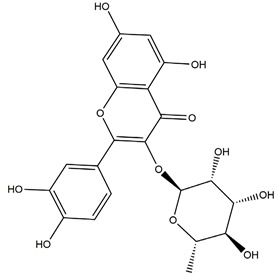	*Solidago chilensis* (Brazilian arnica)	Ethanol/HCl-induced gastric ulcer in Swiss mice	0.46 mg/kg (p.o.)	Inactive	[45]
				1.38 mg/kg (p.o.)	Active	
Afzelin (kaempferol 3-*O*-glucorhamnoside)	* 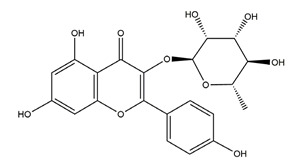 *	*Solidago chilensis* (Brazilian arnica)	Ethanol/HCl-induced gastric ulcer in Swiss mice	0.026 mg/kg (p.o.)	Active	[45]
				0.078 mg/kg (p.o.)		
Hesperidin	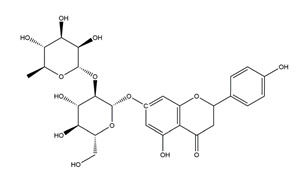	*Citrus sinensis* peel, Citrus fruits	Indomethacin-induced gastric ulcer in Wistar rats	150 mg/kg (i.g.)	Inactive	[47]
				300 mg/kg (i.g.)		
				450 mg/kg (i.g.)	Active	
			Hypothermic restraint stress-induced ulcer in Wistar rats	150 mg/kg (i.g.)	Inactive	
				300 mg/kg (i.g.)	Active	
				450 mg/kg (i.g.)		
			Stress-induced gastric ulcer in diabetic rats	100 mg/kg (i.g.)	Active	[48]
			Ethanol-induced gastric ulcer in Wistar rats	50 mg/kg (p.o.)	Active	[83]
Hypolaetin-8-glucoside	* 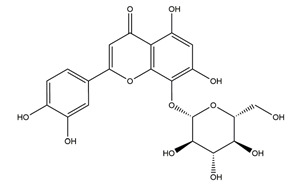 *	*Sideritis leucantha*	Ethanol-induced gastric ulcer in Wistar rats	60 mg/kg (s.c.)	Active	[49]
				80 mg/kg (s.c.)		
				100 mg/kg (s.c.)		
				100 mg/kg (p.o.)	Inactive	
				200 mg/kg (p.o.)	Active	
				300 mg/kg (p.o.)		
Meciadanol (*O*-methyl-3(+)-catechin)	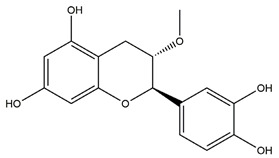	Disconfirmation	Ethanol- induced gastric ulcer in rats; South Indian ulcerogenic diet- gastric ulcer in rats; rice bran oil-induced gastric ulcer in pylorus-ligated rats	150 mg/kg (p.o.)	Active	[50]
Diosmetin	* 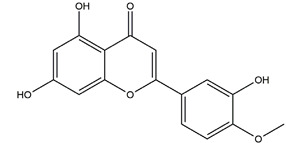 *	*Urera baccifera*	Ethanol-induced gastric ulcer in Wistar rats	3 mg/kg extract (p.o.)	Inactive	[52]
				30 mg/kg extract (p.o.)	Active	
				300 mg/kg extract (p.o).		
Apigenin glucuronide	* 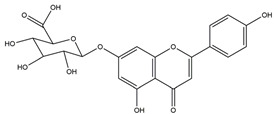 *	*Urera baccifera*	Ethanol-induced gastric ulcer in Wistar rats	3 mg/kg extract (p.o.)	Inactive	[52]
				30 mg/kg extract (p.o.)	Active	
				300 mg/kg extract (p.o.)		
Vexibinol	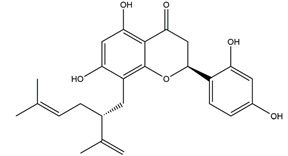	Sophara	HCl-ethanol, 0.6 N HCl 0.2 N NaOH, absolute ethanol and 1% NH_3_-induced gastric ulcers in Wistar rats	100 mg/kg (p.o.)	Active	[53]
				300 mg/kg (p.o.)		
Isoliquiritigenin (4,2’.4’-trihydroxychalcone)	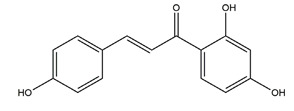	*Glycyrrhiza glabra*	Indomethacin-induced gastric ulcer in ICR mice	100 mg/kg (p.o.)	Active	[56]
			HCI/ethanol-, NaOH-induced gastric ulcer in Sprague-Dawley rats	10 mg/kg (p.o.)	Active	[58]
Chrysin	* 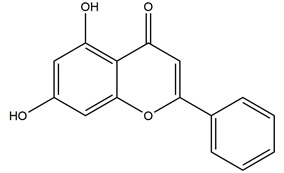 *	Honey, propolis, and various plants	Indomethacin-induced gastric ulcer in Sprague-Dawley rats	50 mg/kg (p.o.)	Active	[57]
				100 mg/kg (p.o.)	Active	
2’,4’-dihydroxychalcone	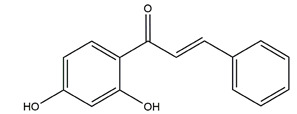	Disconfirmation	HCI/ethanol-, NaOH-, water-immersion stress-induced gastric ulcer in Sprague-Dawley rats	10 mg/kg (p.o.)	Active	[58]
Genistein	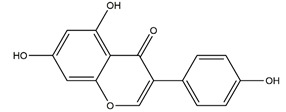	Soy	Indomethacin-induced gastric ulcer in albino rats	10 mg/kg (p.o.)	Active	[62]
Diosmin (diosmetin 7-*O*-rutinoside)	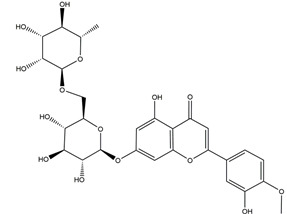	Citrus fruits	Ethanol-induced gastric ulcer in Wistar rats	100 mg/kg (p.o.)	Active	[63]
			70% ethanol-induced gastric ulcer in Sprague-Dawley rats	Chitosan-coated PLGA nanoparticles dispersion at a dose equivalent to 100 mg/kg of diosmin (p.o.)	Active	[123]
Nobiletin (5,6,7,8,3;4”-hexamethoxy flavone)	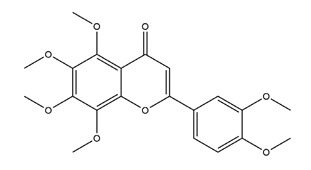	*Aurantii fructus immaturus* citrus fruits	Ethanol-induced gastric ulcer in Kunming mice	5 mg/kg (p.o.)	Active	[64]
				10 mg/kg (p.o.)		
				20 mg/kg (p.o.)		
			Ethanol-induced gastric ulcer in Wistar rats	10 mg/kg (p.o.)	Active	[117]
				25 mg/kg (p.o.)		
				50 mg/kg (p.o.)		
			Aspirin-induced gastric ulcer in Wistar rats	50 mg/kg (p.o.)		
Ternatin (4’-dihydroxy-3,7,8,3’-Tetramethoxyflavone)	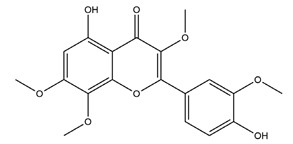	*Egletes viscosa* Less	Ethanol-induced gastric ulcer in Swiss mice	25 mg/kg (p.o.)	Active	[70]
				50 mg/kg (p.o.)		
			Indomethacin-induced gastric ulcer in Swiss mice	25 mg/kg (p.o.)	Inactive	
				50 mg/kg (p.o.)		
			Stress-induced gastric ulcer in Swiss mice	25 mg/kg (p.o.)	Inactive	
				50 mg/kg (p.o.)		
Garcinol	* 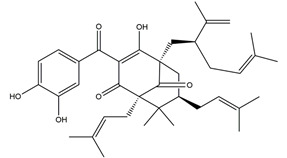 *	*Garcinia indica*	Indomethacin-induced gastric ulcer in Wistar/Crj rats	200 mg/kg (p.o.)	Active	[71]
Anthocyanins (cyanidin-3-glucoside and cyanidin-3-rutinoside: 1:1.5 (*w*/*w*))	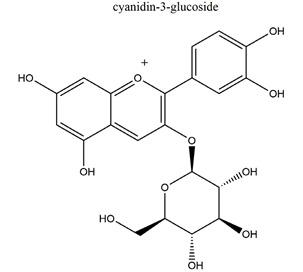 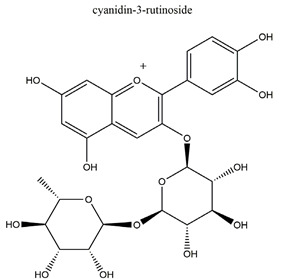	*Rubus coreanus*	Naproxen-induced gastric ulcer in Sprague-Dawley rats	20 mg/kg (p.o.)	Active	[81]
				50 mg/kg (p.o.)		
				80 mg/kg (p.o.)		
Isoorientin	* 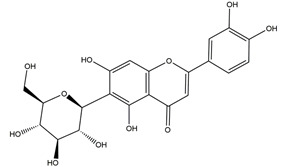 *	*Eremurus* *spectabilis* Bieb.	Indomethacin-induced gastric ulcer in Wistar rats	50 mg/kg (p.o.)	Active	[82]
				100 mg/kg (p.o.)		
				250 mg/kg (p.o.)		
				500 mg/kg (p.o.)		
Aromadendrin-4′-*O*-methyl-ether	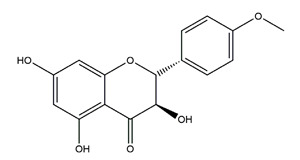	Brazilian green propolis	Ethanol/HCl-induced ulcer in Swiss mice	0.3 mg/kg (p.o.)	Inactive	[84]
				3 mg/kg (p.o.)	Active	
				10 mg/kg (p.o.)		
				30 mg/kg (p.o.)		
			Indomethacin-induced ulcer in Swiss mice	30 mg/kg (p.o.)	Active	
Kaempferide	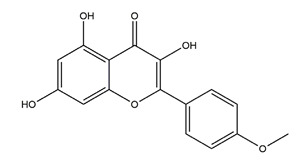	Brazilian green propolis	Ethanol/HCl-induced ulcer in Swiss mice	0.3 mg/kg (p.o.)	Inactive	[84]
				3 mg/kg (p.o.)	Active	
				10 mg/kg (p.o.)		
				30 mg/kg (p.o.)		
			Indomethacin-induced ulcer in Swiss mice	30 mg/kg (p.o.)	Active	
Biochanin A (5,7-Dihydrox -4’-methoxyisoflavone)	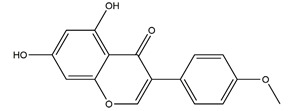	Soy and red clover	Ethanol-induced gastric ulcer in Sprague-Dawley rats	25 mg/kg (p.o.)	Active	[85]
				50 mg/kg (p.o.)		
Silymarin	* 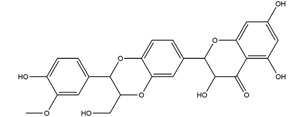 *	*Silybum marianum* (milk thistle) plant	Indomethacin-induced gastric ulcer in albino rats	50 mg/kg (p.o.)	Active	[89]
Kaempferol (3,5,7,4′-tetrahydroxy flavone)	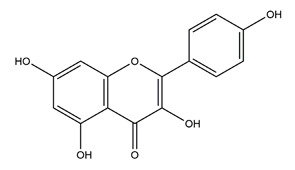	Edible plants (e.g., tea, broccoli) and botanical products	Ethanol-induced gastric ulcer in ICR mice	40 mg/kg (p.o.)	Active	[93]
				80 mg/kg (p.o.)		
				160 mg/kg (p.o.)		
			Ethanol/HCl-induced gastric ulcer in mice	3 mg/kg (p.o.)	Active	[104]
				30 mg/kg (p.o.)		
Rutin (quercetin-3-*O*-rutinoside)	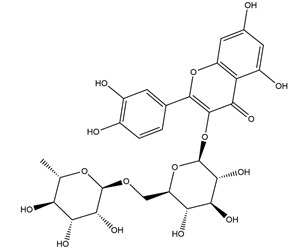	*Ruta graveolens*	Ischemia reperfusion-induced gastric ulcers in Sprague-Dawley rats	50 mg/kg (p.o.)	Active	[94]
				100 mg/kg (p.o.)		
				200 mg/kg (p.o.)		
			Indomethacin-induced gastric ulcer in Wistar albino rats	200 mg/kg (p.o.)	Active	[95]
Marmin (7-(6;7”-dihydroxygeranyloxy) coumarin)	* 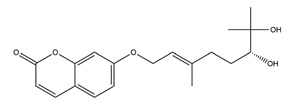 *	*Aurantii fructus immaturus*	Ethanol-induced gastric ulcer in Wistar rats	10 mg/kg (p.o.)	Active	[117]
				25 mg/kg (p.o.)		
				50 mg/kg (p.o.)		
			Aspirin-induced gastric ulcer in Wistar rats	50 mg/kg (p.o.)	Active	

Annotation: p.o.: per os; i.g.: intragastric injection; s.c.: subcutaneous injection.

**Table 2:** Studies on the treatment of peptic ulcer with flavonoids.

Substance	Structure	Sources	Experimental assay	Dose	Activity	Ref.
Catechins	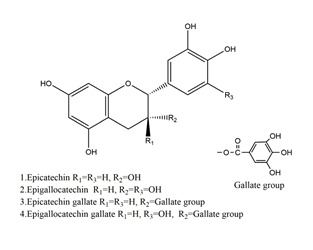	Tea	Ischemia reperfusion-induced gastric ulcers in Sprague-Dawley rats	50 mg/kg (p.o.)	Active	[41]
			Acetic acid-induced gastric ulcer in Sprague-Dawley strain SPF rats	1 mL/100 g (p.o.)	Active	[55]
			*H. pylori-*infected Mongolian gerbils	0.5% Catechin diet (p.o.)	Active	[111]
				1.0% Catechin diet (p.o.)		
				2.0% Catechin diet (p.o.)		
Chalcone	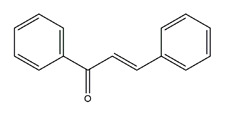	Various plants	H^+^K^+^-ATPase activity	IC50 = 4.8 × 10^–5^M (in vitro)	Active	[46]
Sofalcone	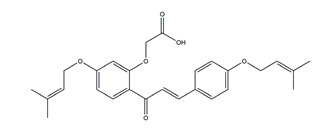	A synthetic derivative of sophoradine	H^+^K^+^-ATPase activity	IC50 = 1.5 × 10^–5^M (in vitro)	Active	[46]
			Consecutive outpatients with peptic ulcer and *H. pylori* infection	Sofalcone (100 mg), rabeprazole (10 mg), clarithromycin (200 mg), and amoxicillin (750 mg) (twice daily for 7 days) (p.o.)	Active	[118]
Sophoradine	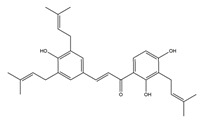	*Sophora subprostrata* root	H^+^K^+^-ATPase activity	IC50= 7.4 × 10^–7^M (in vitro)	Active	[46]
Hypolaetin-8-glucoside	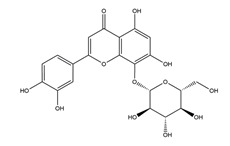	*Sideritis leucantha*	Acetylsalicylic acid (ASA)-induced gastric ulcers in Wistar rats	100 mg/kg (s.c.)	Active	[49]
Hesperidin	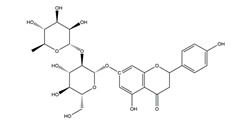	Citrus fruits	Acetic acid-induced chronic gastric ulcer in Wistar rats	1 mg/kg (p.o.)	Inactive	[54]
				3 mg/kg (p.o.)	Active	
				10 mg/kg (p.o.)		
2’,4’-dihydroxychalcone	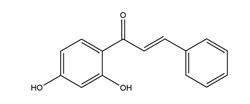	Disconfirmation	Acetic acid-induced gastric ulcer in Sprague-Dawley rats	10 mg/kg (p.o.)	Active	[58]
Garcinol	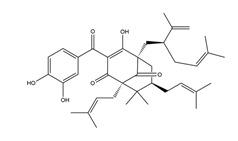	*Garcinia indica*	Stress-induced gastric ulcer in Wistar/Crj rats	200 mg/kg (p.o.)	Active	[71]
Quercetin (combined with famotidine)	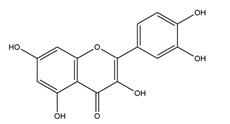	*Madhuca indica J. F. Gmel.* (Sapotaceae), fruits and vegetables	Indomethacin-induced gastric ulcer in albino rats	12 mg/kg famotidine beads and 50 mg/kg quercetin (p.o.)	Active	[77]
Quercetin (3,5,7,3′,4′- Pentahydroxy flavone)	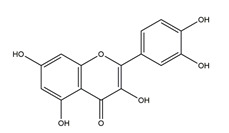	*Madhuca indica J. F. Gmel.* (Sapotaceae), fruits and vegetables	Acetic acid-induced gastric ulcer in Wistar rats	2.5 mg/kg (p.o.)	Inactive	[79]
				5 mg/kg (p.o.)	Active	
				10 mg/kg (p.o.)		
			*H. pylori*-induced gastric ulcer in guinea pigs	200 mg/kg (p.o.)	Active	[112]
			Antibacterial activity (*H. pylori* 26695, *H. pylori* 51, *H. pylori* SS1)	2.5 mM	Inactive	[113]
				5 mM		
				10 mM	Inactive (active for *H. pylori* SS1)	
				20 mM	Active (inactive for *H. pylori* 51)	
Rutin (quercetin-3-*O*-rutinoside)	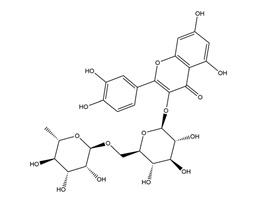	Buckwheat, *Ruta graveolens*	Ethanol-induced gastric ulcers in Wistar rats	20 mg/kg (p.o.)	Active	[80]
				40 mg/kg (p.o.)		
				80 mg/kg (p.o.)		
			Acetic acid-induced gastric ulcers in Wistar rats	20 mg/kg (p.o.)	Active	
				40 mg/kg (p.o.)		
				80 mg/kg (p.o.)		
			Stress-induced gastric ulcers in Wistar rats	20 mg/kg (p.o.)	Active	
				40 mg/kg (p.o.)		
				80 mg/kg (p.o.)		
Epigallocatechin gallate (EGCG)	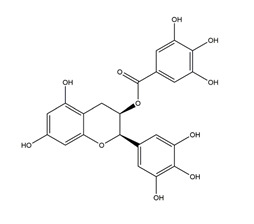	Tea	Indomethacin-induced gastric ulcer in Swiss albino mice	2 mg/kg (p.o.)	Active	[99]
			Killing assay for antibacterial activity (*H. pylori* 110)	Minimum inhibitory concentration (for 50% of isolates): 8 μg/mL (in vitro)	Active	[111]
Epicatechin gallate	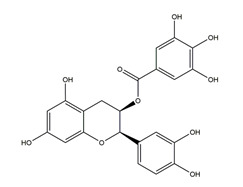	Tea	Killing assay for antibacterial activity (*H. pylori* 55)	Minimum inhibitory concentration (for 50% of isolates): 16 μg/mL (in vitro)	Active	[111]
Epigallocatechin	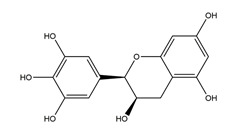	Tea	Killing assay for antibacterial activity (*H. pylori* 55)	Minimum inhibitory concentration (for 50% of isolates): 64 μg/mL (in vitro)	Active	[111]
Epicatechin	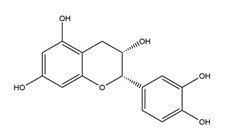	Tea	Killing assay for antibacterial activity (*H. pylori* 55)	Minimum inhibitory concentration (for 50% of isolates): 256 μg/mL (in vitro)	Active	[111]
Theaflavin	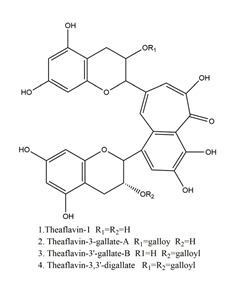	Tea	Killing assay for antibacterial activity (*H. pylori* 55)	Minimum inhibitory concentration (for 50% of isolates): 32 μg/mL (in vitro)	Active	[111]
7-*O*-Butylnaringenin	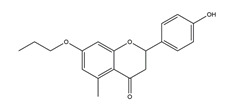	A novel flavonoid modified from naringenin	Antibacterial activity (*H. pylori* 26695, *H. pylori* 51, *H. pylori* SS1)	2.5 mM (in vitro)	Inactive	[113]
				5 mM (in vitro)	Active	
				10 mM (in vitro)		
				20 mM (in vitro)		
Kaempferol (3,5,7,4′-tetrahydroxy flavone)	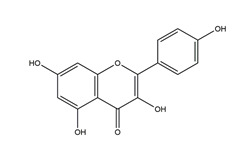	*Kaempferia galanga* L	Antibacterial activity (*H. pylori* 26695, *H. pylori* 51, *H. pylori* SS1)	2.5 mM (in vitro)	Inactive	[113]
				5 mM (in vitro)	Active (inactive for *H. pylori* 51)	
				10 mM (in vitro)	Active	
				20 mM (in vitro)		
Luteolin	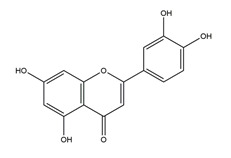	Resedaceae plants	Antibacterial activity (*H. pylori* 26695, *H. pylori* 51, *H. pylori* SS1)	2.5 mM (in vitro)	Inactive	[113]
				5 mM (in vitro)	Active (inactive for H. pylori SS1)	
				10 mM (in vitro)	Active	
				20 mM (in vitro)		
Naringenin	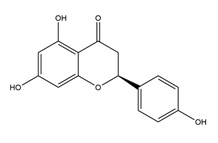	Grapefruits (*Citrus paradise*)	Antibacterial activity (*H. pylori* 26695, *H. pylori* 51, *H. pylori* SS1)	2.5 mM (in vitro)	Inactive	[113]
				5 mM (in vitro)	Active	
				10 mM (in vitro)		
				20 mM (in vitro)		
Hesperetin	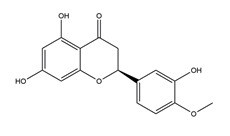	Citrus maxima peel	Antibacterial activity (*H. pylori* 26695, *H. pylori* 51, *H. pylori* SS1)	2.5 mM (in vitro)	Inactive	[113]
				5 mM (in vitro)	Active	
				10 mM (in vitro)		
				20 mM (in vitro)		
Vestitol	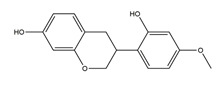	Licorice	Anti-*H. pylori* activity by disk method (*H. pylori*: ATCC43504, ATCC43526, ZLM1007, GP98)	Minimum inhibitory concentration: 12.5 μg/mL (in vitro)	Active	[114]
Licoricone	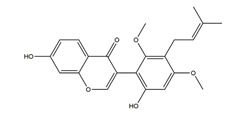	Licorice	Anti-*H. pylori* activity by disk method (*H. pylori*: ATCC43504, ATCC43526, ZLM1007, GP98)	Minimum inhibitory concentration: 12.5 μg/mL (in vitro)	Active	[114]
1-Methoxyphaseollidin	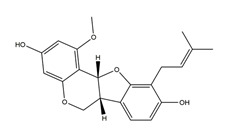	Licorice	Anti-*H. pylori* activity by disk method (*H. pylori*: ATCC43504, ATCC43526, ZLM1007, GP98)	Minimum inhibitory concentration: 16 μg/mL (in vitro)	Active	[114]
Gancaonol C	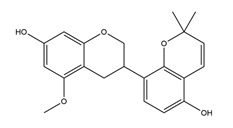	Licorice	Anti-*H. pylori* activity by disk method (*H. pylori*: ATCC43504, ATCC43526, ZLM1007, GP98)	Minimum inhibitory concentration: 16 μg/mL (in vitro)	Active	[114]
Glycyrin	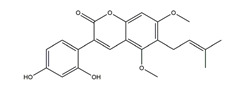	Licorice	Anti-*H. pylori* activity by disk method (*H. pylori*: ATCC43504, ATCC43526, ZLM1007, GP98)	Minimum inhibitory concentration: 50 μg/mL (in vitro)	Active	[114]
Formononetin	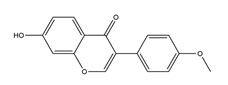	Licorice	Anti-*H. pylori* activity by disk method (*H. pylori*: ATCC43504, ATCC43526, ZLM1007, GP98)	Minimum inhibitory concentration: > 100 μg/mL (in vitro)	Active	[114]
Isolicoflavonol	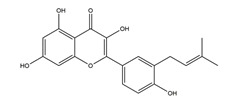	Licorice	Anti-*H. pylori* activity by disk method (*H. pylori*: ATCC43504, ATCC43526, ZLM1007, GP98)	Minimum inhibitory concentration: 25 μg/mL (in vitro)	Active	[114]
Glyasperin D	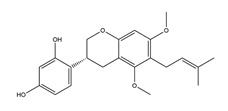	Licorice	Anti-*H. pylori* activity by disk method (*H. pylori*: ATCC43504, ATCC43526, ZLM1007, GP98)	Minimum inhibitory concentration: 25 μg/mL (in vitro)	Active	[114]
6,8-Diprenylorobol	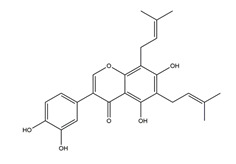	Licorice	Anti-*H. pylori* activity by disk method (*H. pylori*: ATCC43504, ATCC43526, ZLM1007, GP98)	Minimum inhibitory concentration: 50 μg/mL (in vitro)	Active	[114]
Gancaonin I	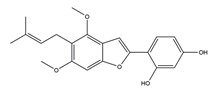	Licorice	Anti-*H. pylori* activity by disk method (*H. pylori*: ATCC43504, ATCC43526, ZLM1007, GP98)	Minimum inhibitory concentration: 50 μg/mL (in vitro)	Active	[114]
Dihydrolicoisoflavone A	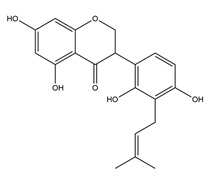	Licorice	Anti-*H. pylori* activity by disk method (*H. pylori*: ATCC43504, ATCC43526, ZLM1007, GP98)	Minimum inhibitory concentration: 25 μg/mL (in vitro)	Active	[114]
Gancaonol B	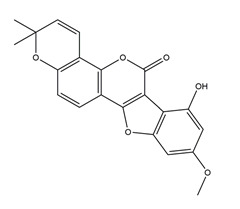	Licorice	Anti-*H. pylori* activity by disk method (*H. pylori*: ATCC43504, ATCC43526, ZLM1007, GP98)	Minimum inhibitory concentration: 32 μg/mL (in vitro)	Active	[114]
Isorhamnetin (quercetin 3-methyl ether)	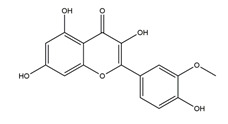	*Cistus laurifolius*	Anti-*H. pylori* activity by agar dilution method (*H. pylori*: NCTC11637)	Minimum inhibitory concentration: 3.9 μg/mL (in vitro)	Active	[115]
Quercetin 3,7-dimethyl ether	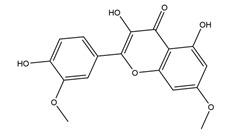	*Cistus laurifolius*	Anti-*H. pylori* activity by agar dilution method (*H. pylori*: NCTC11637)	Minimum inhibitory concentration: 62.5 μg/mL (in vitro)	Active	[115]
Kaempferol 3,7-dimethyl ether	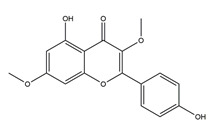	*Cistus laurifolius*	Anti-*H. pylori* activity by agar dilution method (*H. pylori*: NCTC11637)	Minimum inhibitory concentration: 62.5 μg/mL (in vitro)	Active	[115]
Irisolidone	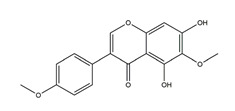	*Pueraria thunbergiana* (Leguminosae)	Growth inhibition assay of *H. pylori* (*H. pylori*: ATCC43504, NCTC11637, NCTC11638, 82516, 82548, 4)	Minimum inhibitory concentration: 12.5–25 μg/mL (in vitro)	Active	[116]
Tectorigenin	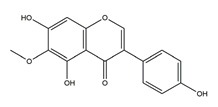	*Pueraria thunbergiana* (Leguminosae)	Growth inhibition assay of *H. pylori* (*H. pylori*: ATCC43504, NCTC11637, NCTC11638, 82516, 82548, 4)	Minimum inhibitory concentration: 100 μg/mL (in vitro)	Active	[116]
Genistein	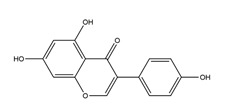	*Pueraria thunbergiana* (Leguminosae)	Growth inhibition assay of *H. pylori* (*H. pylori*: ATCC43504, NCTC11637, NCTC11638, 82516, 82548, 4)	Minimum inhibitory concentration: > 100 μg/mL (in vitro)	Active	[116]

Annotation: p.o.: per os; s.c.: subcutaneous injection.

## Abbreviations

BabA: blood group antigen adhesin; bFGF: basic fibroblast growth factor; CAT: catalase; CD31: platelet endothelial cell adhesion molecule-1; COX: cyclooxygenase; DPPH: 1,1-Diphenyl-2-picrylhydrazyl radical; EGCG: epigallocatechin gallate; ERK: extracellular-signal-regulated kinases; GPx: glutathione peroxidase; GSH: glutathione; *H. pylori*: *Helicobacter pylori*; H_2_O_2_: hydrogen peroxide; HO-1: Heme oxygenase-1; IBD: inflammatory bowel disease; IL-10: interleukin-10; IL-1β: interleukin-1β; IL-6: interleukin-6; JNK: c-Jun *N*-terminal kinases; MAPK: mitogen-activated protein kinases; MCP-1: monocyte chemotactic protein-1; MDA: malondialdehyde; MMP-2: metalloproteinase-2; MPO: myeloperoxidase: NFκB: nuclear factor kappa B; NO: Nitric oxide; Nrf2: nuclear factor erythroid 2-related factor 2; NSAIDs: non-steroidal anti-inflammatory drugs; O2: superoxide anion radical; OH.: hydroxyl radical; OipA: outer inflammatory protein adhesin; p38: p38 mitogen-activated protein kinases; PGE2: prostaglandin E2; PGs: prostaglandins; PLGA: chitosan-poly (d,l-lactide-co-glycolide); PPI: proton pump inhibitor; ROS: reactive oxygen species; SOD: superoxide dismutase; TNF-α: tumor necrosis factor-α; VEGF: vascular endothelial growth factor
